# Overcoming the Obstacle of Polymer–Polymer Resistances in Double Layer Solid Polymer Electrolytes

**DOI:** 10.1021/acs.jpclett.1c00366

**Published:** 2021-03-12

**Authors:** Christofer Sångeland, Trine Tjessem, Jonas Mindemark, Daniel Brandell

**Affiliations:** Department of Chemistry - Ångström Laboratory, Uppsala University, Lägerhyddsvägen 1, SE-751 21 Uppsala, Sweden

## Abstract

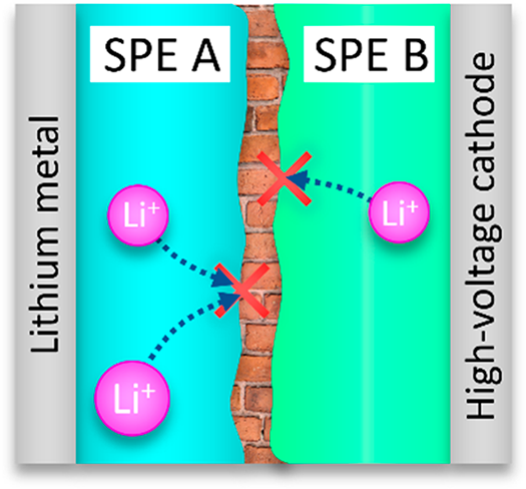

Double-layer solid polymer electrolytes (DLSPEs) comprising one layer that is stable toward lithium metal and one which is stable against a high-voltage cathode are commonly suggested as a promising strategy to achieve high-energy-density lithium batteries. Through in-depth EIS analysis, it is here concluded that the polymer–polymer interface is the primary contributor to electrolyte resistance in such DLSPEs consisting of polyether-, polyester-, or polycarbonate-bad SPEs. In comparison to the bulk ionic resistance, the polymer–polymer interface resistance is approximately 10-fold higher. Nevertheless, the interfacial resistance was successfully lowered by doubling the salt concentration from 25 to 50 wt % LiTFSI owing to improved miscibility at the interface of the two polymer layers.

Combining a lithium metal anode with a high-voltage cathode will enable lithium batteries with energy densities beyond 260 Wh kg^–1^.^1^ Unfortunately, uncontrolled electrolyte degradation render existing liquid electrolytes incompatible with lithium metal.^2,3^ To avoid these stability issues, one viable alternative is to replace the liquid electrolyte with a solid polymer electrolyte (SPE).^4^ It has been shown, however, to be highly challenging to design a homogeneous SPE with a sufficiently wide electrochemical stability window (ESW) and passivating abilities toward both the lithium metal anode and a high-voltage cathode.^5^ In fact, most SPE battery cells tested in the scientific literature are benchmarked against low-potential LiFePO_4_.^5^ A promising solution would be a so-called double-layer solid polymer electrolyte (DLSPE), i.e., a laminate architecture comprising one polymer electrolyte that exhibits anodic stability and one layer that exhibits cathodic stability, effectively widening the ESW of the SPE. This concept has already been successfully demonstrated by, for example, Goodenough et al.; a poly(ethylene oxide)–poly(*N*-methyl-malonic amide)-based DLSPE operating at 65 °C was used in a lithium metal battery with a LiCoO_2_ cathode.^6^ Similarly, Zhou et al. have developed a DLSPE consisting of a cross-linked poly(ethylene oxide)-based anolyte and a poly(oxalate)-based catholyte which was used with a LiNi_0.6_Mn_0.2_Co_0.2_O_2_ cathode.^7^ A similar outcome was achieved by creating a multilayered SPE where the middle layer facilitated fast conduction of lithium ions and the outer layers in contact with the anode and cathode, respectively, contained components for favorable solid electrolyte interphase (SEI) and cathode electrolyte interphase (CEI) formation.^8^ Given the myriad of different polymer electrolytes readily available and the relatively simplistic design of DLSPEs,^5^ a rapid development in this field can be predicted in the foreseeable future.

One of the reasons for poly(ethylene oxide) (PEO) dominating the field of SPEs is because it forms a stable SEI with lithium metal.^5,9^ In spite of this, advancements have been partially hampered by the low oxidation onset of PEO, between 3.8–4.2 V vs Li+/Li according to voltammetry techniques, hence ruling out the implementation of high-voltage cathodes.^10−14^ While alternative SPE host materials such as poly(ε-caprolactone) (PCL) and poly(trimethylene carbonate) (PTMC) have exhibited oxidation onsets spanning from 4.5–5 V vs Li^+^/Li,^15,16^ there are indications of them being less stable against lithium metal compared to PEO.^17^ Accordingly, by combining PEO with either PTMC or PCL to form a DLSPE, the ESW can be significantly extended. However, any such DLSPE approach will introduce yet another significant interface in the battery cell, e.g., that between the two different polymer layers. Given that lithium transport across interfaces is generally known to be a severe bottleneck in solid-state LIBs,^18−21^ this is a potential bottleneck in the DLSPE cell architecture as well. As such, correct interpretation and understanding of the polymer–polymer interface in different materials systems are essential, but this is currently lacking in the literature.

To this end, we studied the polymer–polymer interfaces in SPE cells spanning the range of polyether, polyester, and polycarbonate-based electrolyte materials using electrochemical impedance spectroscopy (EIS). SPEs consisting of high-molecular-weight PEO, PCL, and PTMC with 25 wt % LiTFSI were assembled into DLSPEs via hot pressing (see the Supporting Information for experimental details). Henceforth, the different SPEs and DLSPEs are denoted according to the polymer host(s) and LiTFSI salt concentration, e.g., PEO:25 and PEO–PCL:25. The resulting films were sandwiched between two stainless steel blocking electrodes and characterized using EIS. The real and imaginary impedance–frequency response of the DLSPEs can be seen in the Nyquist plots in Figure 1. The interpretation of these data is not straightforward, but upon initial inspection, one common feature present in all three DLSPEs is the existence of two semicircles, indicating the presence of two different processes occurring at separate time scales.

**Figure 1:**
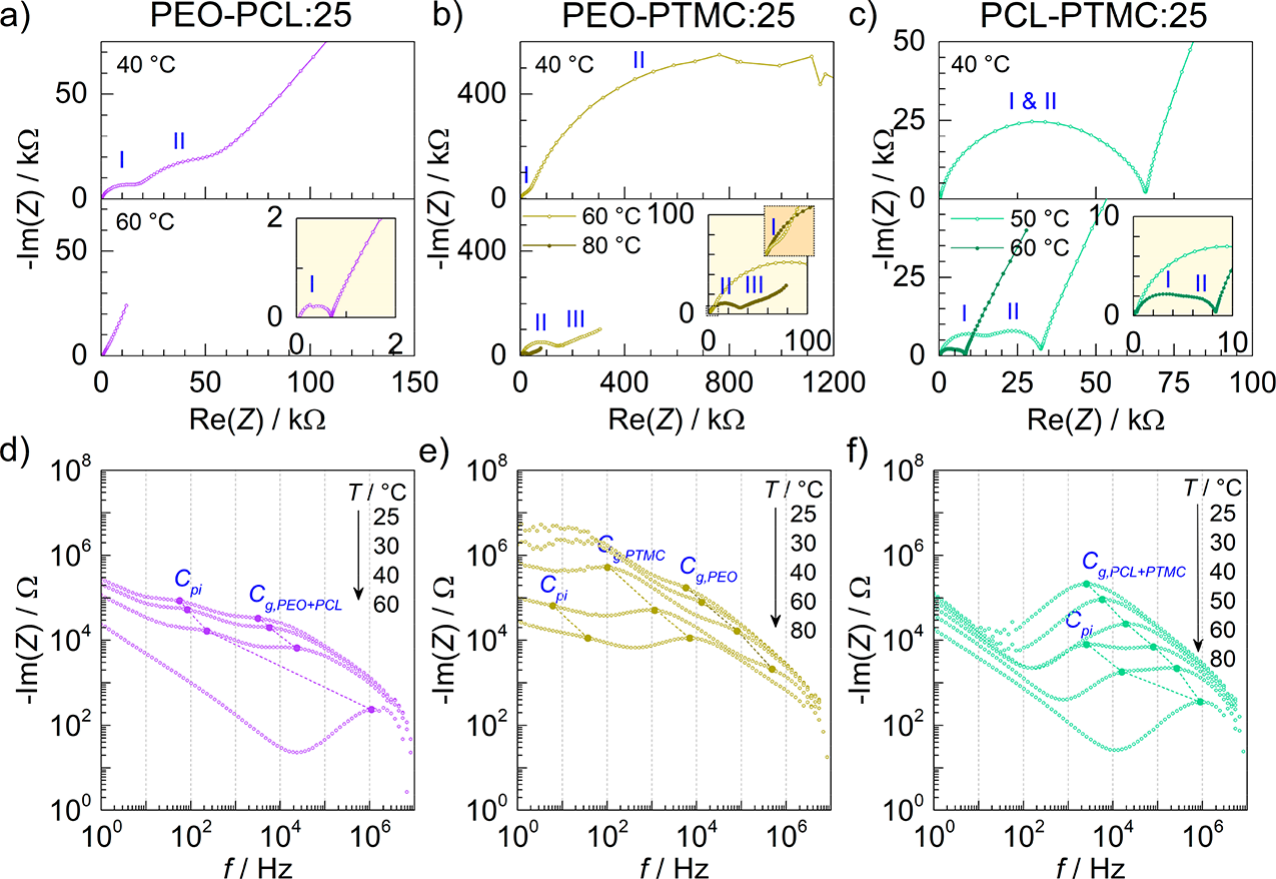
Nyquist and Bode plots of (a, d) PEO–PCL, (b, e) PEO–PTMC, and (c, f) PCL–PTMC DLSPEs with 25 wt % LiTFSI at temperatures ranging from 25 to 80 °C. Local maxima associated with the capacitive processes of the polymer–polymer interface (*C*_pi_) and bulk electrolytes (*C*_g_) have been marked in the Bode plots. At certain temperatures, the features belonging to the polymer interface and the bulk electrolyte become indistinguishable.

In the case of PEO–PCL:25, one small (I) and one midsized (II) semicircle are clearly visible at 40 °C, see Figure 1a. Following heating to 60 °C, the impedance–frequency response was reduced to a single semicircle (I). Similarly, PCL–PTMC:25 also exhibited two semicircles, albeit only at 50 and 60 °C, see Figure 1c. This indicates that the processes associated with the bulk electrolyte and the interface exhibit different temperature dependence. Consequently, the two processes are distinguishable when their time constants deviate, which happens to be at 50 and 60 °C. In PEO–PTMC:25, one small (I) and one much larger (II) semicircle are observed at high and intermediate frequencies at 40 °C, see Figure 1b. An additional third semicircle (III) emerges at low frequencies when the temperature is increased to 60 °C. When the temperature reaches 80 °C, only two semicircles at mid and low frequencies remain visible (II and III). Unlike PEO–PCL:25 and PCL–PTMC:25, the tail corresponding to the charging of the double layer at the electrodes and ionic diffusion is not fully resolved for PEO–PTMC:25, indicating that diffusion processes lay outside of the frequency range of the measurement.

Based on the Nyquist plots in Figure 1a–c alone, it is difficult to identify what the semicircles represent with certainty. Hence, the imaginary response (−Im(*Z*)) was plotted against the frequency (*f*) in Bode plots in order to identify the characteristic frequencies associated with each semicircle, see Figure 1d–f. For reference, the impedance–frequency response was also measured for single-layer SPEs with 25 wt % LiTFSI, as well as DLSPEs consisting of the same material in both layers (e.g., PEO–PEO:25), see Figures S1 and S2. As is evident in Figure 1d, two local maxima, situated between 60 to 300 and 2 × 10^4^ to 10^6^ Hz at 25 to 40 °C, are observed in PEO–PCL:25. The local maximum between 2 × 10^4^ to 10^6^ Hz is also observed in PEO:25 and PCL:25 (Figures S1d and S1e) and correspond to the geometric capacitances (*C*_g_). Hence, the local maxima located at lower frequencies between 60 and 300 Hz seen in Figure 1d must stem from new feature, i.e., the polymer–polymer interface (*C*_pi_) in PEO–PCL:25. A small shift in relaxation frequencies is expected given changes in film thickness which in turn effects both resistance and capacitance. The same reasoning was applied on PEO–PTMC:25 and PCL–PTMC:25 in order to identify the polymer–polymer interface. Notably, *C*_pi_ could not be observed in the reference DLSPEs consisting of the same SPEs (i.e., PEO–PEO:25, PCL–PCL:25, and PTMC–PTMC:25) either, see Figure S2.

Using the Bode plots, it was thus possible to construct equivalent circuits to simulate the impedance–frequency response of the different SPE and DLSPE configurations, see Figure 2. Single-layer SPEs, such as PEO:25, were modeled using a Debye equivalent circuit consisting of a constant phase element (CPE_g_, _PEO_) in parallel with a resistor (*R*_PEO_ or *R*_b_) and an additional CPE element (CPE_dl_), see circuit A. CPE_g_,_PEO_ represents the geometric capacitance of PEO:25, *R*_PEO_ (or *R*_b_) represents the bulk resistance of the SPE, and CPE_dl_ represents the double layer capacitance at the polymer–electrode interface. The geometric and double layer capacitances are modeled using constant phase elements (CPEs) instead of regular capacitors to account for the roughness of the electrode surface. At temperatures above the melting point of semicrystalline SPEs, the response from the geometric capacitance shifts to frequencies exceeding the measurement range as the ionic conductivity increases, and the Debye circuit is effectively reduced to a resistor (*R*_b_) in series with constant phase element (CPE_dl_), similar to liquid electrolytes. This is why, for example, PEO:25 and PCL:25 do not exhibit a semicircle at 60 °C, see Figures S1a and S1b. Hence, the remaining semicircle in Figure 1a at 60 °C must originate from the polymer–polymer interface. To model the polymer–polymer interface between PEO–PCL:25 and PCL–PTMC:25, an additional resistor (*R*_pi_) in parallel with a CPE (CPE_pi_) was added between the bulk polymer resistance (*R*_PEO+PCL_) and CPE_dl_, see circuits B and D; *R*_pi_ and CPE_pi_ represent the ionic resistance and capacitance of the polymer–polymer interface, respectively. Since the impedance contributions arising from the individual layers in PEO–PCL:25 and PCL–PTMC:25 were indistinguishable, due to overlapping time constants, the resistance and geometric capacitance for each layer were merged in the equivalent circuit. This is necessary to avoid the equivalent circuit from falsely assigning resistance contributions that cannot be accurately separated.

**Figure 2:**
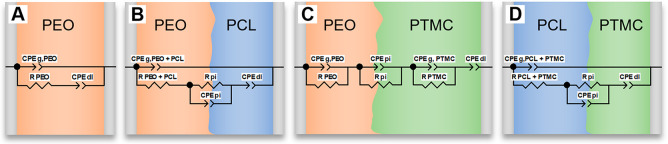
Equivalent circuits used to model impedance–frequency response of SPEs (represented by PEO) and DLSPEs with 25 wt % LiTFSI.

In the case of PEO–PTMC:25 (circuit C), three separate semicircles were observed at 60 °C; hence, a separate circuit consisting of three parallel circuits was devised to model the impedance–frequency response. We attribute this behavior to the large difference in frequency range associated with the geometric capacitance for PEO:25 and PTMC:25, see Figures S1d and S1f. The large difference is due to the high resistance of PTMC:25, which in turn affects the time constant of the system, shifting the impedance response to lower frequencies.^5^ The high resistance of PTMC:25 is attributed to its high glass transition temperature in comparison to PEO:25 (−11 and −36 °C, respectively), see Figure S3.^15,22^

Using the equivalent circuits in Figure 2, it is possible to determine *R*_b_ in the single-layer SPEs and *R*_b_ and *R*_pi_ in the DLSPEs (among other parameters); see Tables S1–S9. In ascending order, the *R*_pi_ at 60 °C was 0.4, 1.9, and 628.7 kΩ for PEO–PCL:25, PCL–PTMC:25, and PEO–PTMC:25, respectively. This goes to show that the lithium transport from one polymer host to another is highly dependent on the compatibility of the two SPEs. Ensuring compatibility between the SPE systems therefore becomes essential, so as not to compromise performance.

The total ionic conductivity values calculated from the total resistance (including the interfacial resistance) are shown in Figure 3a. The ionic conductivities for PEO:25, PCL:25, and PTMC:25 range from 10^–8^ to 10^–3^ S cm^–1^ at temperatures from 25 to 90 °C and agree well with previously published data.^15,23−25^ As expected, the rapid increase in ionic conductivity exhibited by PEO:25 and PCL:25 between 50 and 60 °C and 40 and 50 °C, respectively, coincide with the melting points (*T*_m_) of PEO:25 and PCL:25 at 55 and 43 °C, respectively; see Figure S3. With a comparatively high *T*_g_ (Figure S3), translating into low polymer chain mobility, the ionic conductivity of PTMC:25 is lower in comparison to PEO:25 and PCL:25. PTMC:25 is also completely amorphous and does, therefore, not display the same jump in ionic conductivity as PEO:25 and PCL:25 do at the melting point.

**Figure 3:**
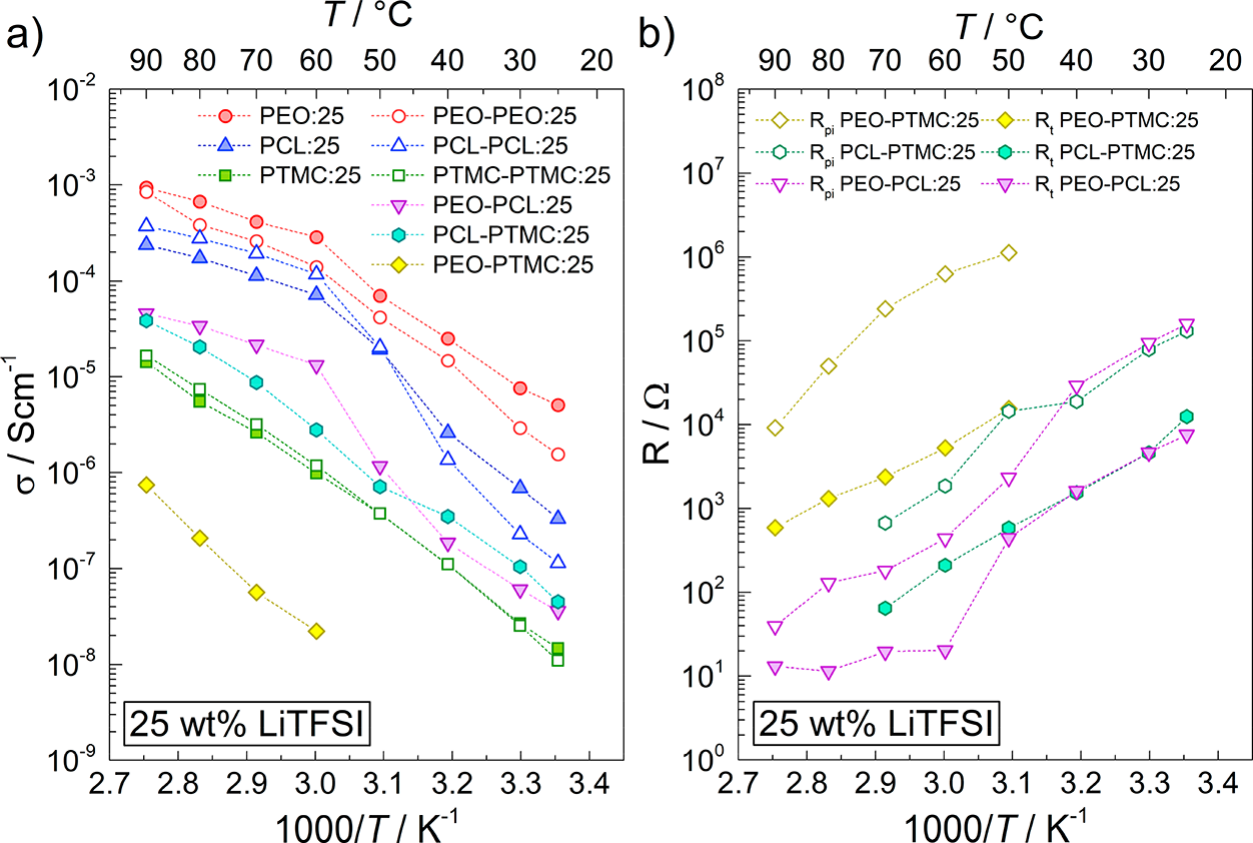
(a) Total ionic conductivity of the SPEs and DLSPEs with 25 wt % LiTFSI at temperatures ranging from 25 to 90 °C. (b) Bulk polymer resistance normalized by DLSPE thickness and multiplied by 10 μm (*R*_t_) compared to the polymer interfacial resistance (*R*_pi_) at temperatures ranging from 25 to 90 °C.

In the case of PEO–PCL:25 and PEO–PTMC:25, the ionic conductivity of the DLSPEs was significantly lower in comparison to the single-layer SPEs, indicating a massive increase in resistance due to the polymer–polymer interface. In the worst-case scenario, the ionic conductivity of PEO–PTMC:25 was approximately 10^4^-fold and 10^2^-fold worse relative to the ionic conductivities of PEO:25 and PTMC:25, respectively. In contrast, PCL–PTMC:25 exhibited ionic conductivity values between those of PCL:25 and PTMC:25, indicating negligible impact on the SPE resistance overall. Interestingly, the ionic conductivities of PEO–PEO:25, PCL–PCL:25, and PTMC–PTMC:25 are similar to those of the single-layer SPEs, showing that the substantial polymer–polymer interfacial resistance is observed only in DLSPEs consisting of different SPEs, see Figure 3a.

From an application point of view, minimizing the SPE thickness is necessary in real battery cells to compensate for the relatively low ionic conductivity.^5^ To illustrate such a scenario, bulk polymer resistance was normalized by the thickness of the DLSPE and then multiplied by 10 μm to simulate a 10 μm-thick DLSPE (*R*_t_), see Figure 3b. Since the cross-sectional area remains constant and independent of DLSPE thickness, this allows for direct comparison with the interfacial resistance values. The absence of a defined thickness for the interface prevents calculation of an interfacial ionic conductivity for direct comparison with the bulk ionic conductivity. It is seen that *R*_pi_ would completely dominate the resistance of the DLSPE in this realistic scenario. As this example illustrates, reducing the interfacial resistance substantially by ensuring SPE–SPE compatibility is a necessary step toward realizing practical DLSPEs.

Based on the aforementioned observations, it is evident that the polymer–polymer interface gives rise to a hefty resistance within the DLSPE that completely dominates the total resistance of the cell. A plausible explanation to the origin of the polymer–polymer interface resistance is the difference in coordination strength between the polyether-, polyester-, and polycarbonate-based polymer hosts, which could give rise to a detrimental concentration gradient across the interface.^26^ Alternatively, the immiscibility of two different polymer layers could also hinder lithium transport across the interface. For example, when poly(ethylene carbonate) (PEC) and PTMC are blended together instead of fabricated as a DLSPE, two distinct glass transition temperatures (*T*_g_) are observed, indicating that PEC and PTMC form separate domains.^27^ However, when the LiTFSI salt concentration is increased from 10 to 100 mol % for these samples, the two glass transitions belonging to PEC and PTMC become less pronounced and slowly merge, suggesting gradual miscibility of PEC and PTMC facilitated by their mutual affinity for coordinating to Li^+^ cations. Inspired by this finding, the LiTFSI salt concentration was doubled from 25 to 50 wt % in the DLSPEs to facilitate interfacial miscibility and thereby potentially reduce interfacial resistance.

The impedance–frequency response of the DLSPEs and SPEs with 50 wt % LiTFSI can be seen in Figures 4 and S4, respectively. The ionic properties of PEO–PCL:50 could be extracted using circuit B. In the case of PEO–PTMC:50 and PCL–PTMC:50, *R*_b_ and *R*_pi_ could be distinguished only in a limited temperature interval because of overlapping time constants for these processes. Outside this interval, only the total resistance was determined using circuit A. The estimated values for *R*_b_ and *R*_pi_ can be seen in Tables S10–S15.

**Figure 4:**
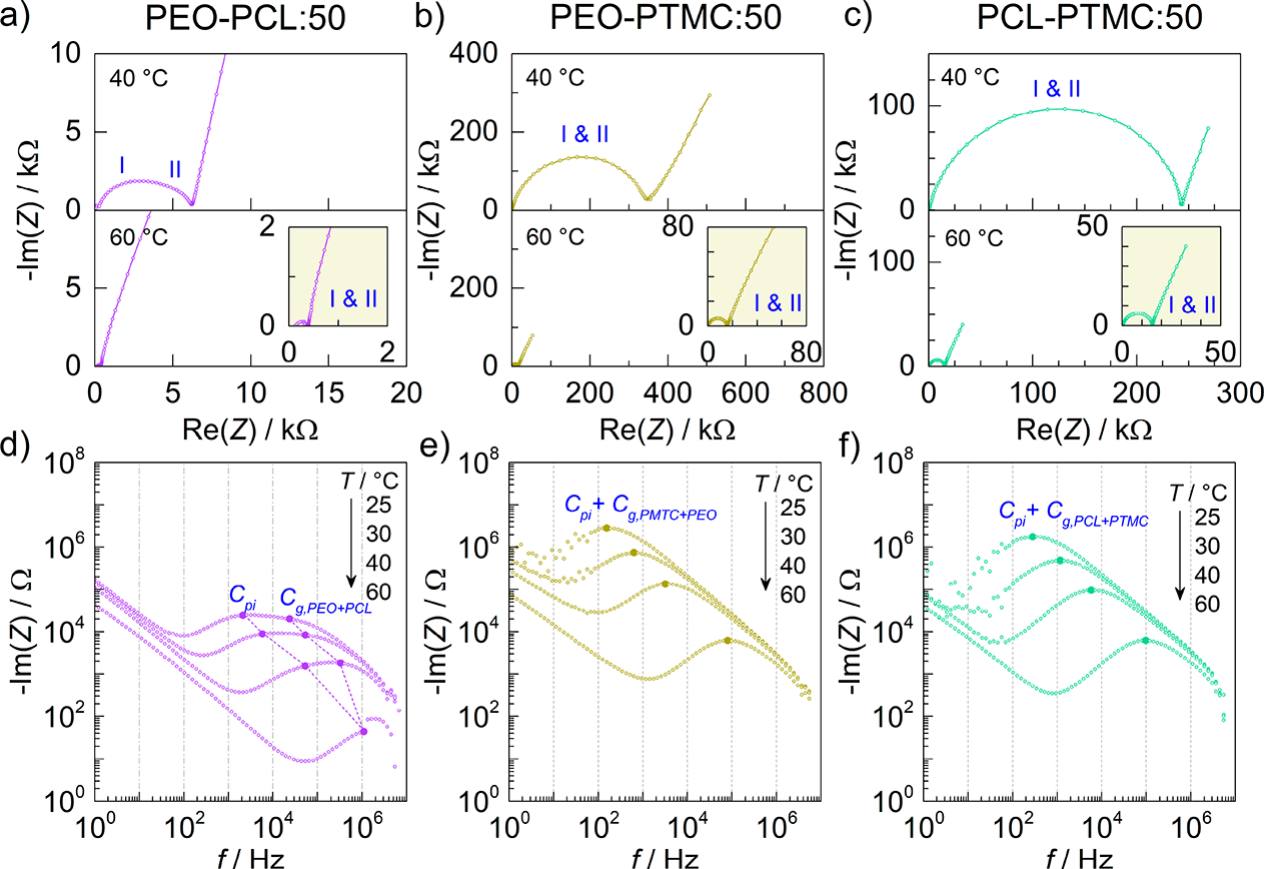
Nyquist and Bode plots of (a, d) PEO–PCL, (b, e) PEO–PTMC, and (c, f) PCL–PTMC with 50 wt % LiTFSI at temperatures ranging from 25 and 60 °C. Local maxima corresponding to the relaxation frequencies of the constant phase elements belonging to the polymer interface (*C*_pi_) and bulk electrolyte (*C*_g_) have been marked in the Bode plots. In certain cases, the features belonging to the polymer interface and the bulk electrolyte become indistinguishable.

The total ionic conductivity of the SPEs and DLSPEs with 50 wt % are shown in Figure 5a. In contrast with the 25 wt % data, both PEO:50 and PCL:50 show responses typical of amorphous SPEs, resulting in increased conductivity in the lower temperature range.^28,29^ The suppression of crystallinity is also evidenced by differential scanning calorimetry, see Figure S3. In contrast, conductivity of PTMC is reduced at the higher salt concentration due to stiffening of the polymer host from bridging between Li^+^ and coordination sites on the polymer chain.^30,31^ The ionic conductivity of PEO–PCL:50 and PEO–PTMC:50 was considerably improved relative to that of PEO:50, PCL:50, and PTMC:50, see Figure 5a, and PEO–PCL:25 and PEO–PTMC:25, see Figure S5. As seen in Figure 5b, this can be attributed to a 10^2^-fold and 10-fold reduction of *R*_pi_ in PEO–PTMC and PEO–PCL, respectively, when the LiTFSI concentration is doubled from 25 to 50 wt %. Furthermore, at elevated temperatures (>60 °C) the ionic conductivity of PEO–PCL:50 appears to be limited by the ionic conductivity of PCL:50 instead of the polymer–polymer interface, see Figure 5a. In the case of PCL–PTMC:50 on the other hand, the ionic conductivity was lower in comparison to PCL–PTMC:25. As observed in Figure 3a, the limiting component of PCL–PTMC:25 is the high resistance of PTMC:25 and not the polymer–polymer interface. In fact, increasing the salt concentration to 50 wt % increases the resistance of the polymer–polymer interface. Hence, any improvement in the ionic conductivity in PCL:50 is dwarfed by the low ionic conductivity of PTMC:50 and the increase in *R*_pi_.

**Figure 5:**
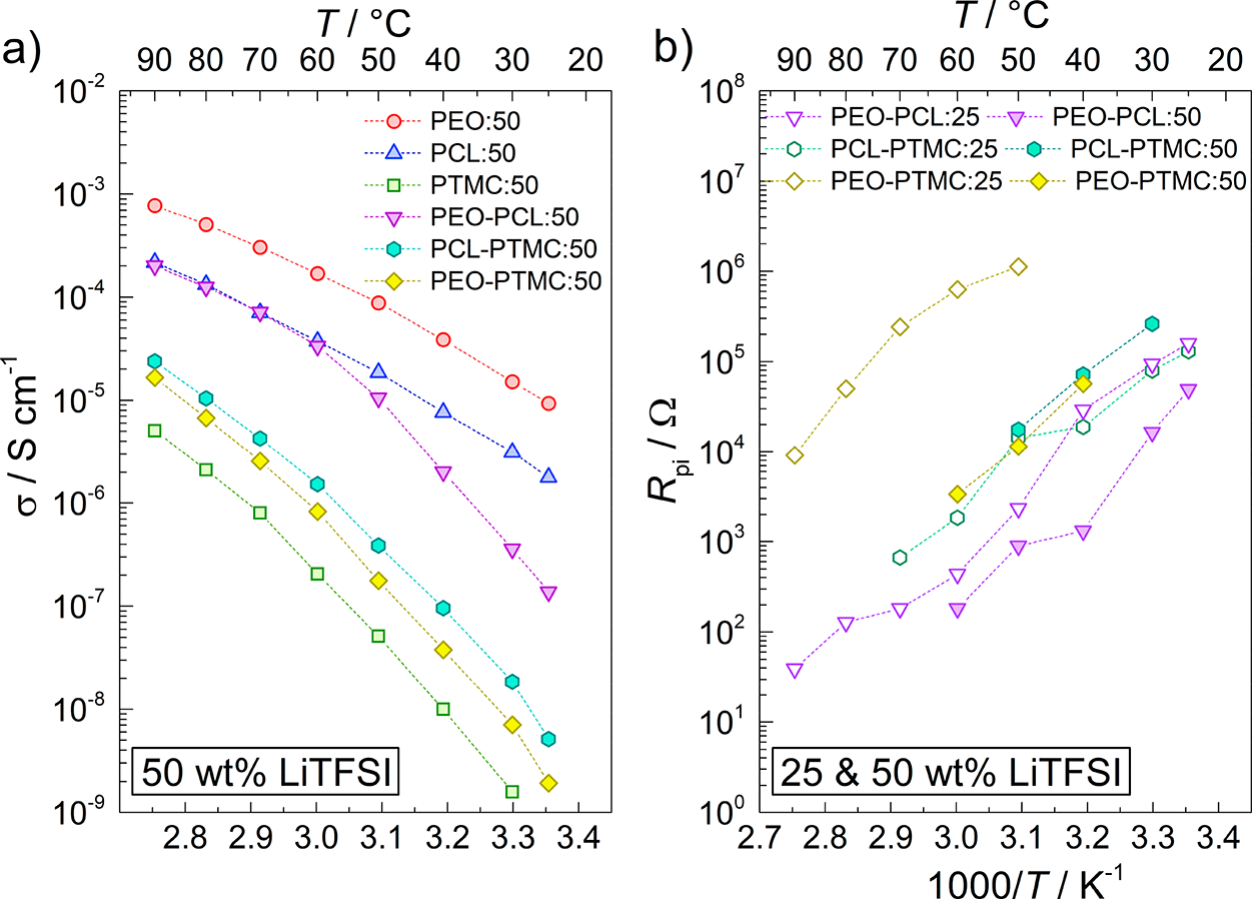
(a) Total ionic conductivity of the SPEs and DLSPEs with 50 wt % LiTFSI at temperatures ranging from 25 to 90 °C. (b) Comparison of polymer interfacial resistances (*R*_pi_) in DLSPEs with 25 and 50 wt % LiTFSI.

This goes to show that the polymer–polymer resistivity resulting from the DLSPE approach—in contrast to what has been previously claimed—can have detrimental effects on the overall electrolyte performance, also when using commonly employed SPE polymer hosts. The EIS analysis renders it possible to separate the bulk SPE from the interfacial resistance contributions, where then up to a 10^4^-fold reduction in ionic conductivity was observed. Through the choice of materials, there is a clear tendency that the chemical compatibility between the two polymers employed controls the severity of this problem. However, we could also show that straightforward strategies can be employed to overcome these effects, here exemplified by simply increasing the lithium salt concentration. Other strategies to increase the miscibility and reduce the resistance can also be envisioned. While EIS studies can pinpoint problematic aspects of this interfacial resistance, the ionic transport over the induced barrier needs to be understood better by means of, for example, computational simulations and spectroscopic techniques such as NMR. Intuitively and based on the results presented here, a chemical compatibility between the polymer layers seems to be crucial for mitigating the observed resistance. With a better understanding of the interfacial ion transport and considering that both SPE layers are soft materials, it should eventually be possible to also chemically tailor this interface to facilitate ion transport across it.

## Supporting Information Available

Experimental section, Nyquist and Bode plots of SPEs and reference DLSPEs, parameters obtained from equivalent circuit modeling, thermal properties of SPEs with 0, 25, and 50 wt % salt, and SEM micrographs of DLSPEs (PDF)
