# Supplementary material for: Overcoming the Obstacle of Polymer–Polymer Resistances in Double Layer Solid Polymer Electrolytes
Source: J Phys Chem Lett. 2021 Mar 12;12(11):2809–14. doi: 10.1021/acs.jpclett.1c00366 (PMC8006132; doi:10.1021/acs.jpclett.1c00366)
Supplement: Supplementary file 1 — jz1c00366_si_001.pdf [file jz1c00366_si_001.pdf]

Supporting Information for

Overcoming the Obstacle of Polymer–Polymer Resistances in Double Layer Solid Polymer Electrolytes

Christofer Sångeland, Trine Tjessem, Jonas Mindemark, Daniel Brandell

Table of Contents

1. Experimental methods S2
1.1. Materials S2
1.2. Synthesis of PTMC and PCL S2
1.3. Polymer electrolyte preparation and assembly of the DLSPEs S2
1.4. Electrochemical impedance spectroscopy S2
1.5. Scanning electron microscopy S3
1.6. Differential scanning calorimetry S3
2. Additional results S4
2.1. Nyquist and Bode plots of SPEs and DLSPEs with 25 wt% LiTFSI S4
2.2. Equivalent circuit fitting parameters for SPEs and DLSPEs with 25 wt% LiTFSI S5
2.3. Thermal properties of PEO, PCL and PTMC with different concentrations of salt S8
2.4. Nyquist and Bode plots of SPEs with 50 wt% LiTFSI S9
2.5. Equivalent circuit fitting parameters for SPEs and DLSPEs with 50 wt% LiTFSI S10
2.6. Total ionic conductivity of DLSPEs S11
2.7. Morphology of the polymer–polymer interface in DLSPEs S12
3. References S12

1. Experimental methods

1.1. Materials

Lithium bis(triflouromethanesulfonyl imide) (LiTFSI; BASF), acetonitrile (ACN; Sigma Aldrich, 99.8%, anhydrous), tetrahydrofuran (THF; Sigma Aldrich, 99.8%, anhydrous), poly(ethylene oxide) (PEO; Sigma Aldrich, M_w: 2 000 000 g mol^{-1}), trimethylene carbonate (TMC; Richman), ε-caprolactone (CL; Perstorp), stannous 2-ethylhexanoate ($Sn(Oct)_2$; Sigma 95 %), toluene (Acros Organics, 99.8%, Super dry with molecular sieves).

1.2. Synthesis of PTMC and PCL

High molecular weight poly(trimethylene carbonate) (PTMC) and poly(ε-caprolactone) (PCL) were synthesized using a method described elsewhere.[1] Described briefly, 200 mmol of TMC or CL monomer were added together with 40 µL of 1M $Sn(Oct)_2$ catalyst in toluene solution in a stainless steel reactor. All constituents, including the reactor, were dried prior to the synthesis in order to remove traces of water. The TMC and CL monomers were dried by distillation over CaH_2 under reduced pressure. The reactor was then placed in an oven set at 130 °C for 72 h. The contents were thoroughly mixed by shaking the reactor every 30 min during the first 90 min in the oven. After the polymerization, the reactor was allowed to cool and the final product could be divided into small pieces for later use. In the case of PTMC, the obtained product was transparent and rubbery, and according to previous gel permeation chromatography (GPC) measurements, the M_w was approximately 185 000 – 334 000 g mol^{-1}. In contrast, synthesized PCL was opaque, milky white in color, solid and had a M_w of 740 000 g mol^{-1}. Despite differences in M_w, PEO, PCL and PTMC can still be compared, since ionic conductivity and transference numbers are expected to remain constant above 100 000 g mol^{-1}.[2,3] All steps were carried out in argon atmosphere.

1.3. Polymer electrolyte preparation and assembly of the DLSPEs

The single-layer solid polymer electrolyte films were prepared by first dissolving polymer and LiTFSI salt by heating and stirring overnight. PEO was dissolved in THF, while PCL and PTMC were dissolved in ACN. The ratio of polymer to solvent was 0.02 g mL^{-1} for PEO and 0.0267 g mL^{-1} for PCL and PTMC. The quantity of salt is expressed relative to the total weight of the SPE i.e. polymer and salt. The solutions were subsequently cast in PTFE molds 30 mm in diameter (dried at 60 °C for 12 hours prior to use) and placed in an oven connected to an external pump. The solvent was evaporated using a method described elsewhere.[4] In short, the pressure in the oven was lowered to 200 mbar and kept at 30 °C for 20 hours after which the pressure was lowered to approximately 1.5 mbar and the temperature was increased to 60 °C and held for an additional 40 h. Next, the resulting films were placed between two PTFE sheets and heated to 80 °C for 30 min in an MTI 6T hydraulic lamination hot press. After 30 minutes of heating, the films were hot pressed at 25 MPa for an additional 30 min at the same temperature. The pressure was retained until a temperature of 40 °C was reached after the heater had been turned off. Similarly, the double-layer solid polymer electrolytes were assembled by placing two single layer films (after hot pressing) between two PTFE sheets and heating at 50 °C for 20 min followed by pressing at 1 MPa for 25 mins. Once again, the pressure was maintained until the temperature was 40 °C after the heater had been turned off. Using this method, it was possible to manufacture DLSPEs with a well-defined interface with no gaps, see Figure S6. Next, 13 mm in diameter films were punched out and their thickness was determined using a Mitituyo digital indicator micrometer. Finally, the films were hermetically sealed in 2025 coin cells (Hohsen, dried at 60 °C for 60 hours prior) along with a PTFE spacer ring. All steps were carried out in argon atmosphere.

1.4. Electrochemical impedance spectroscopy

The impedance–frequency response of the polymer electrolyte films was studied using electrochemical impedance spectroscopy (EIS). Prior to measurement, the polymer films were annealed at an elevated temperature to ensure good contact between the electrolyte and the stainless-steel blocking electrodes. The impedance-frequency response was measured between 10^7 and 1 Hz with an amplitude of 50 mV at temperatures ranging from 25 to 90 °C using a Schlumberger impedance/Gain-Phase analyzer SI 1260. The bulk resistance of the single layer polymer electrolyte films was determined by simulating impedance response using a Debye equivalent circuit in ZView v.3.2b (Scribner Associates).[4]

1.5. Scanning electron microscopy

Morphological characterization of the polymer-polymer interface was done using a Carl Zeiss Merlin scanning electron microscope (SEM) with a beam current of 100 pA and an acceleration voltage of 3 kV. Cross-sections of the DLSPEs were prepared by cutting with a scalpel. All steps were done under argon atmosphere.

1.6. Differential scanning calorimetry

The glass transition temperatures and melting points of the polymer electrolyte films was determined using differential scanning calorimetry (DSC) on a TA instrument DSC Q2000. Polymer electrolyte samples were hermitically sealed in aluminum pans and then cooled to −80 °C at 5 °C min^{-1} followed by thermal equilibration. Next, samples were heated to 100 °C at 10 °C min^{-1} and once again allowed to thermally equilibrate, after which the thermal cycle was repeated. Finally, glass transition temperatures and melting points were determined from the second heating cycle using TA instruments Universal Analysis 2000 v. 4.5A.

2. Additional results

2.1. Nyquist and Bode plots of SPEs and DLSPEs with 25 wt% LiTFSI

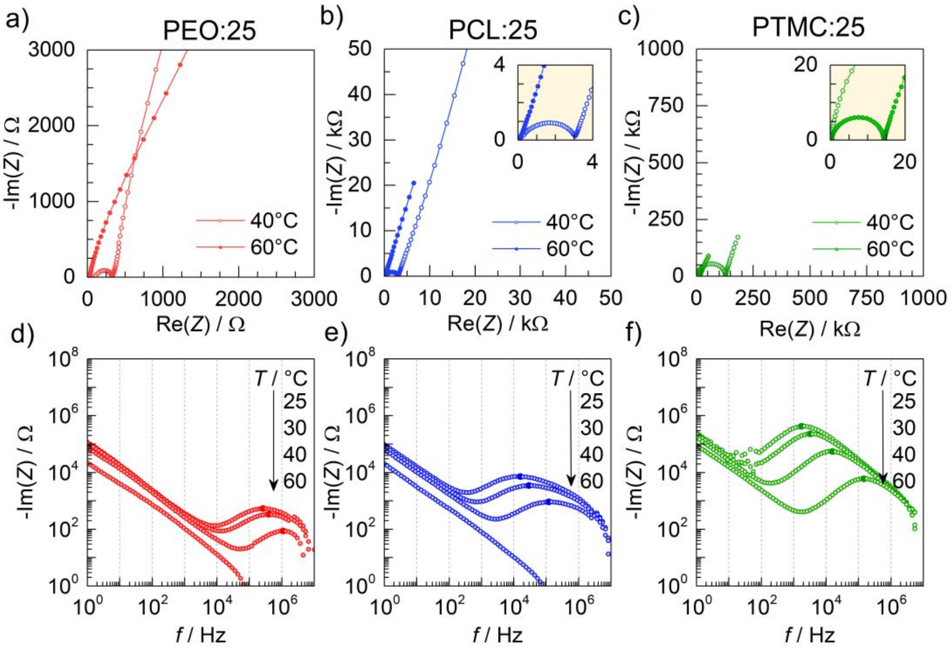

Figure S1. Nyquist and Bode plots of PEO:25 (red), PCL:25 (blue) and PTMC:25 (green) at temperatures ranging from 25 to 60 °C. The local maximum corresponding to the relaxation frequency for ion transport in the bulk polymer electrolyte have been marked in the Bode plots.

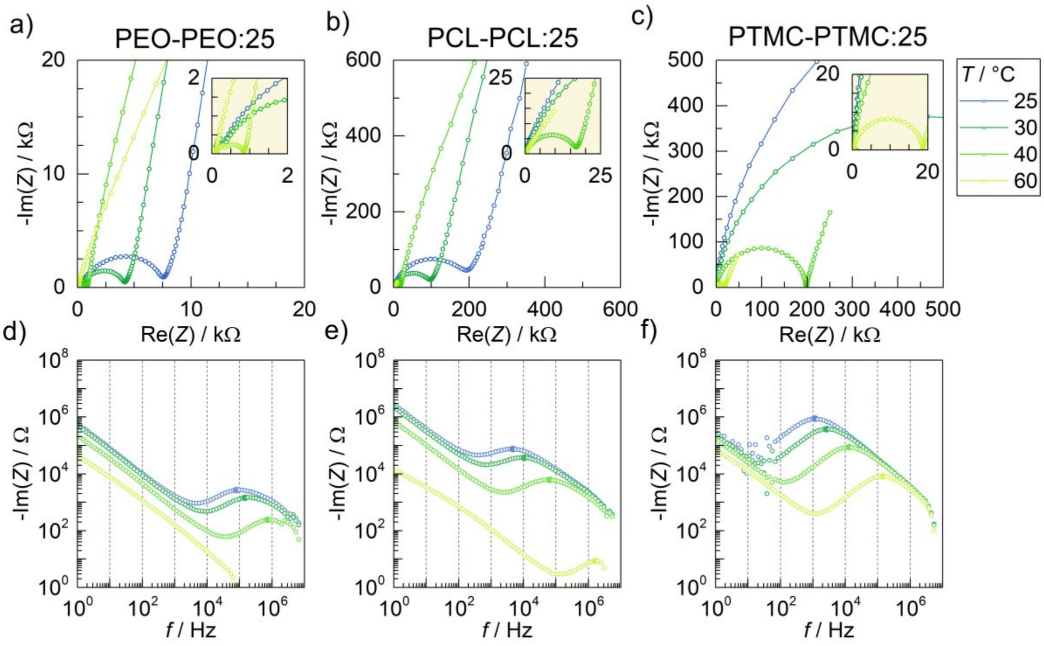

Figure S2. Nyquist and Bode plots of DLSPEs consisting of the same SPEs with 25 wt% LiTFSI at temperatures ranging from 25 to 60 °C. The legend for all figures is positioned on the right.

2.2. Equivalent circuit fitting parameters for SPEs and DLSPEs with 25 wt% LiTFSI

Table S1. Equivalent circuit fitting parameters for PEO with 25 wt% LiTFSI at temperatures ranging from 25 to 90 °C. The thickness and area of the film was 114 µm and 1.33 cm^2, respectively. The error percentages are listed in parentheses and errors exceeding 10% are marked in red.

T / K	R_{PEO} / Ω		$CPE_{g, PEO}$ / F s^{1-p}		CPE_{dl} / F s^{1-p}		σ / S cm^{-1}
298.15	1688	(0.29)	1.02×10^{-8}	(8.12)	1.36×10^{-6}	(1.80)	5.08×10^{-6}
303.15	1130	(0.30)	1.74×10^{-8}	(9.92)	1.47×10^{-6}	(1.52)	7.59×10^{-6}
313.15	340.9	(0.71)	4.49×10^{-8}	(24.55)	1.71×10^{-6}	(3.79)	2.51×10^{-5}
323.15	122.5	(0.41)			2.49×10^{-6}	(1.70)	7.00×10^{-5}
333.15	30.1	(1.19)			4.35×10^{-6}	(3.34)	2.85×10^{-4}
343.15	20.7	(1.50)			6.23×10^{-6}	(4.44)	4.14×10^{-4}
353.15	12.8	(1.99)			7.53×10^{-6}	(5.29)	6.70×10^{-4}
363.15	9.2	(2.53)			9.49×10^{-6}	(5.90)	9.37×10^{-4}

Table S2. Equivalent circuit fitting parameters for PCL with 25 wt% LiTFSI at temperatures ranging from 25 to 90 °C. The thickness and area of the film was 106 µm and 1.33 cm^2, respectively. The error percentages are listed in parentheses.

T / K	R_{PCL} / Ω		$CPE_{g, PCL}$ / F s^{1-p}		CPE_{dl} / F s^{1-p}		σ / S cm^{-1}
298.15	23947	(0.29)	1.39×10^{-8}	(4.11)	2.22×10^{-6}	(0.79)	3.33×10^{-7}
303.15	11534	(0.23)	1.38×10^{-8}	(4.80)	2.65×10^{-6}	(0.56)	6.91×10^{-7}
313.15	3059	(0.17)	8.20×10^{-8}	(8.05)	3.45×10^{-6}	(0.39)	2.61×10^{-6}
323.15	410.2	(0.38)			3.89×10^{-6}	(3.12)	1.94×10^{-5}
333.15	111.2	(0.29)			7.37×10^{-6}	(3.31)	7.17×10^{-5}
343.15	70.26	(0.68)			1.05×10^{-5}	(6.94)	1.13×10^{-4}
353.15	45.9	(0.38)			1.75×10^{-5}	(1.63)	1.74×10^{-4}
363.15	33.41	(0.35)			2.27×10^{-5}	(0.23)	2.39×10^{-4}

Table S3. Equivalent circuit fitting parameters for PTMC with 25 wt% LiTFSI at temperatures ranging from 25 to 90 °C. The thickness and area of the film was 187 µm and 1.33 cm^2, respectively. The error percentages are listed in parentheses.

T / K	R_{PTMC} / Ω		$CPE_{g, PTMC}$ / F s^{1-p}		CPE_{dl} / F s^{1-p}		σ / S cm^{-1}
298.15	958450	(0.38)	1.22×10^{-10}	(8.63)	6.02×10^{-7}	(6.72)	1.47×10^{-8}
303.15	527310	(0.14)	1.69×10^{-10}	(2.85)	8.95×10^{-7}	(1.95)	2.67×10^{-8}
313.15	127000	(0.09)	1.72×10^{-10}	(2.48)	1.18×10^{-6}	(0.88)	1.11×10^{-7}
323.15	37644	(0.09)	1.70×10^{-10}	(3.11)	1.53×10^{-6}	(1.23)	3.74×10^{-7}
333.15	14337	(0.10)	2.21×10^{-10}	(3.26)	2.03×10^{-6}	(1.53)	9.81×10^{-7}
343.15	5334	(0.09)	2.20×10^{-10}	(3.88)	2.86×10^{-6}	(1.64)	2.64×10^{-7}
353.15	2543	(0.15)	3.83×10^{-10}	(7.87)	4.98×10^{-6}	(2.12)	5.53×10^{-6}
363.15	992.1	(0.10)	2.91×10^{-10}	(9.59)	6.78×10^{-6}	(1.73)	1.42×10^{-5}

Table S4. Equivalent circuit fitting parameters for PEO-PCL with 25 wt% LiTFSI at temperatures ranging from 25 to 90 °C. The thickness and area of the film was 120 µm and 1.33 cm^2, respectively. The error percentages are listed in parentheses and errors exceeding 10% are marked in red.

T / K	$R_{PEO+PCL}$ / Ω		$CPE_{g, PEO+PCL}$ / F s^{1-p}		R_{pi} / Ω		CPE_{pi} / F s^{1-p}		CPE_{dl} / F s^{1-p}		σ / S cm^{-1}
298.15	91457	(1.12)	6.37×10^{-9}	(1.19)	158500	(2.69)	8.80×10^{-8}	(3.77)	9.70×10^{-7}	(0.89)	3.33×10^{-7}
303.15	55428	(0.31)	7.14×10^{-9}	(0.93)	94200	(1.52)	9.03×10^{-8}	(2.58)	1.30×10^{-6}	(0.49)	6.91×10^{-7}
313.15	19428	(1.20)	9.02×10^{-9}	(3.77)	29016	(5.24)	1.52×10^{-7}	(13.58)	2.34×10^{-6}	(1.64)	2.61×10^{-6}
323.15	5358	(0.57)	2.82×10^{-8}	(3.40)	2335	(6.00)	5.09×10^{-7}	(20.92)	4.09×10^{-6}	(0.67)	1.94×10^{-5}
333.15	244.1	(4.53)			439.9	(2.62)	1.82×10^{-10}	(18.43)	8.43×10^{-6}	(1.73)	7.17×10^{-5}
343.15	234.4	(2.80)			182	(3.72)	2.40×10^{-10}	(21.97)	1.07×10^{-5}	(2.97)	1.13×10^{-4}
353.15	137.7	(3.20)			128.2	(3.80)	2.91×10^{-11}	(50.51)	2.60×10^{-5}	(3.40)	1.75×10^{-4}
363.15	157.3	(1.48)			39.53	(6.07)	9.62×10^{-11}	(45.81)	2.87×10^{-5}	(2.66)	2.39×10^{-4}

Table S5. Equivalent circuit fitting parameters for PEO-PTMC with 25 wt% LiTFSI at temperatures ranging from 25 to 90 °C. The thickness and area of the film was 219 μm and 1.33 cm^2, respectively. The error percentages are listed in parentheses and errors exceeding 10% are marked in red.

T	R_{PEO}		$CPE_{g, PEO}$		R_{PTMC}		$CPE_{g, PTMC}$		R_{pi}		CPE_{pi}		CPE_{dl}		σ
/ K	/ Ω		/ F s^{1-p}		/ Ω		/ F s^{1-p}		/ Ω		/ F s^{1-p}		/ F s^{1-p}		/ S cm^{-1}
298.15	250780	(6.93)	4.04×10^{-10}	(14.83)	1.26×10^{7}	(4.24)	2.60×10^{-9}	(7.58)							1.28×10^{-9}
303.15	114300	(1.46)	4.74×10^{-10}	(15.15)	6.20×10^{6}	(2.47)	3.30×10^{-9}	(5.80)							2.61×10^{-9}
313.15	24645	(4.97)	4.18×10^{-10}	(14.42)	1.61×10^{6}	(1.50)	6.17×10^{-9}	(4.87)							1.00×10^{-8}
323.15	6404	(2.88)	5.62×10^{-10}	(12.62)	332860	(0.71)	5.27×10^{-9}	(1.82)	1.12×10^{6}	(0.97)	6.11×10^{-7}	(2.09)			1.13×10^{-8}
333.15	1883	(5.33)	1.57×10^{-10}	(29.10)	113440	(0.65)	5.65×10^{-9}	(2.13)	628650	(3.20)	1.44×10^{-6}	(1.67)			2.21×10^{-8}
343.15	361.4	(22.64)	4.48×10^{-11}	(152.18)	51875	(3.76)	5.52×10^{-9}	(13.69)	240760	(39.05)	2.25×10^{-6}	(15.69)	6.74×10^{-6}	(123.05)	5.62×10^{-8}
353.15	347.8	(2.14)			28500	(0.76)	6.77×10^{-9}	(1.88)	49943	(7.25)	2.48×10^{-6}	(4.20)	1.14×10^{-5}	(1.70)	2.09×10^{-7}
363.15	209.9	(1.88)			12796	(0.84)	6.21×10^{-9}	(3.23)	9111	(27.66)	5.36×10^{-6}	(17.65)	1.48×10^{-5}	(1.95)	7.45×10^{-7}

Table S6. Equivalent circuit fitting parameters for PCL-PTMC with 25 wt% LiTFSI at temperatures ranging from 25 to 90 °C. The thickness and area of the film was 308 μm and 1.33 cm², respectively. The error percentages are listed in parentheses and errors exceeding 10% are marked in red.

T	***R*** $_{PCL+PTMC}$		***CPE*** $_{g,\ PCL+PTMC}$		***R*** $_{pi}$		***CPE*** $_{pi}$		***CPE*** $_{dl}$		**σ**
/ K	/ Ω		/ F s^{1-p}		/ Ω		/ F s^{1-p}		/ F s^{1-p}		/ Scm^{-1}
298.15	381670	(16.39)	2.31×10^{-10}	(22.98)	130250	(47.79)	3.79×10^{-10}	(43.35)	1.48×10^{-6}	(2.61)	4.52×10^{-8}
303.15	142110	(28.28)	1.27×10^{-10}	(70.49)	79315	(50.61)	5.32×10^{-10}	(38.10)	1.67×10^{-6}	(0.99)	1.05×10^{-7}
313.15	47210	(1.39)	3.73×10^{-10}	(0.34)	18806	(3.40)	1.22×10^{-10}	(8.10)	2.01×10^{-6}	(2.73)	3.51×10^{-7}
323.15	18126	(0.94)	1.15×10^{-9}	(4.30)	14339	(1.75)	8.72×10^{-9}	(12.56)	2.71×10^{-6}	(6.99)	7.13×10^{-7}
333.15	6470	(6.51)	3.12×10^{-9}	(2.91)	1848	(1.53)	3.88×10^{-9}	(21.66)	2.57×10^{-6}	(21.95)	2.78×10^{-6}
343.15	1980	(25.92)	5.79×10^{-9}	(201.32)	672.9	(75.79)	4.89×10^{-9}	(91.44)	5.15×10^{-6}	(2.98)	8.73×10^{-6}
	R $_{PCL}$		***CPE*** $_{g,\ PCL}$		***R*** $_{pi+PTMC}$		***CPE*** $_{g,\ pi+PTMC}$				
353.15	341.6	(1.71)			775.5	(0.81)	4.84×10^{-10}	(6.26)	7.13×10^{-6}	(2.49)	2.07×10^{-5}
363.15	337.3	(1.66)			262.3	(2.19)	2.10×10^{-10}	(13.13)	1.02×10^{-5}	(2.18)	3.86×10^{-5}

Table S7. Equivalent circuit fitting parameters for PEO-PEO with 25 wt% LiTFSI at temperatures ranging from 25 to 90 °C. The thickness and area of the film was 171 μm and 1.33 cm², respectively. The error percentages are listed in parentheses and errors exceeding 10% are marked in red.

T	***R*** $_{Total}$		***CPE*** $_{g,\ Total}$		***CPE*** $_{dl}$		**σ**
/ K	/ Ω		/ F s^{1-p}		/ F s^{1-p}		/ S cm^{-1}
298.15	8311	(0.33)	3.94×10^{-9}	(6.93)	3.23×10^{-7}	(1.57)	1.55×10^{-6}
303.15	4416	(0.43)	6.86×10^{-9}	(7.37)	3.72×10^{-7}	(1.92)	2.92×10^{-6}
313.15	877.3	(0.59)	1.83×10^{-8}	(18.50)	6.81×10^{-7}	(2.27)	1.47×10^{-5}
323.15	309.4	(0.47)			1.22×10^{-6}	(1.49)	4.17×10^{-5}
333.15	92.39	(0.88)			3.00×10^{-6}	(2.39)	1.40×10^{-4}
343.15	49.72	(1.43)			5.68×10^{-6}	(3.52)	2.59×10^{-4}
353.15	33.62	(1.12)			6.53×10^{-6}	(3.85)	3.83×10^{-4}
363.15	15.26	(1.34)			7.99×10^{-6}	(4.61)	8.45×10^{-4}

Table S8. Equivalent circuit fitting parameters for PCL-PCL with 25 wt% LiTFSI at temperatures ranging from 25 to 90 °C. The thickness and area of the film was 318 μm and 1.33 cm², respectively. The error percentages are listed in parentheses and errors exceeding 10% are marked in red.

T	***R*** $_{Total}$		***CPE*** $_{g,\ Total}$		***CPE*** $_{dl}$		**σ**
/ K	/ Ω		/ F s^{1-p}		/ F s^{1-p}		/ S cm^{-1}
298.15	207840	(0.43)	1.40×10^{-9}	(3.40)	7.80×10^{-8}	(1.23)	1.15×10^{-7}
303.15	104480	(0.43)	1.65×10^{-9}	(4.76)	1.09×10^{-9}	(1.11)	2.29E$\times10^{-7}$
313.15	17535	(0.28)	2.51×10^{-9}	(5.39)	2.75×10^{-7}	(0.69)	1.36×10^{-6}
323.15	1175	(0.76)	1.69×10^{-8}	(21.23)	1.14×10^{-6}	(1.86)	2.04×10^{-5}
333.15	204.6	(1.28)			1.28×10^{-5}	(3.11)	1.17×10^{-4}
343.15	123.9	(1.14)			1.06×10^{-5}	(2.63)	1.93×10^{-4}
353.15	86.5	(1.02)			1.06×10^{-5}	(2.94)	2.77×10^{-4}
363.15	64.13	(1.03)			1.46×10^{-5}	(2.77)	3.73×10^{-4}

Table S9. Equivalent circuit fitting parameters for PTMC-PTMC with 25 wt% LiTFSI at temperatures ranging from 25 to 90 °C. The thickness and area of the film was 292 μm and 1.33 cm², respectively. The error percentages are listed in parentheses and errors exceeding 10% are marked in red.

T	***R*** $_{Total}$		***CPE*** $_{g,\ Total}$		***CPE*** $_{dl}$		**σ**
/ K	/ Ω		/ F s^{1-p}		/ F s^{1-p}		/ S cm^{-1}
298.15	2.00×10^{6}	(0.41)	1.45×10^{-10}	(3.15)	5.90×10^{-7}	(12.77)	1.10×10^{-8}
303.15	871710	(0.17)	1.57×10^{-10}	(1.81)	8.51×10^{-7}	(3.14)	2.52×10^{-8}
313.15	197690	(0.10)	1.43×10^{-10}	(2.31)	1.28×10^{-6}	(0.72)	1.11×10^{-7}
323.15	58477	(0.14)	1.60×10^{-10}	(2.869	1.87×10^{-6}	(0.77)	3.75×10^{-7}
333.15	18548	(0.20)	1.76×10^{-10}	(4.42)	2.98×10^{-6}	(0.90)	1.18×10^{-6}
343.15	6888	(0.23)	1.88×10^{-10}	(6.68)	4.64×10^{-6}	(0.90)	3.19×10^{-6}
353.15	2965	(0.29)	2.82×10^{-10}	(10.00)	6.91×10^{-6}	(0.99)	7.40×10^{-6}
363.15	1318	(0.46)	1.01×10^{-10}	(20.31)	1.05×10^{-5}	(1.46)	1.67×10^{-5}

2.3. Thermal properties of PEO, PCL and PTMC with different concentrations of salt

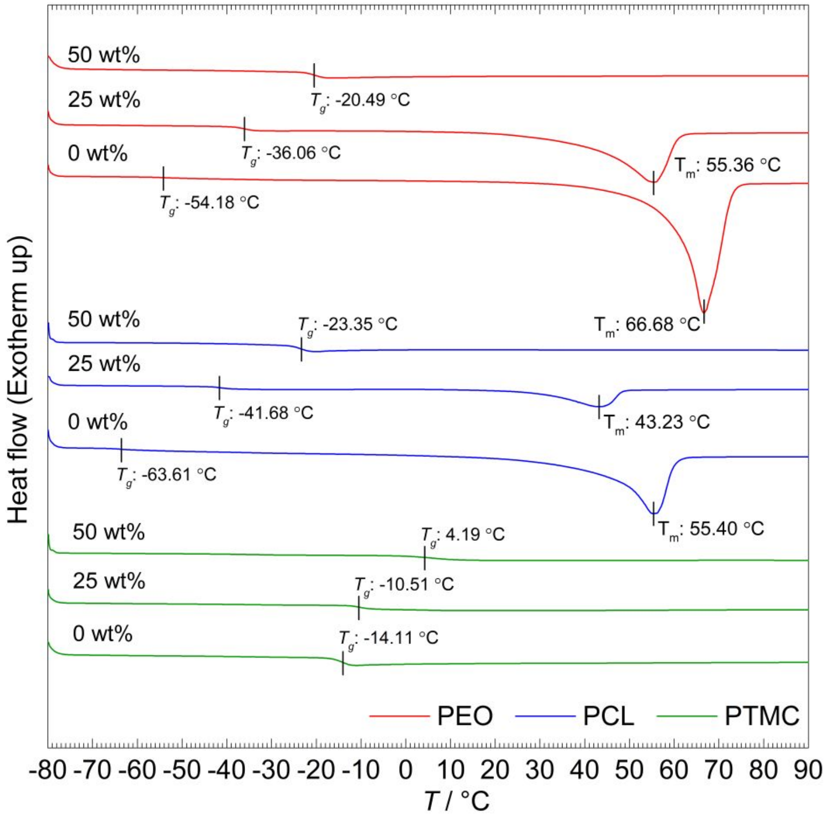

Figure S3. Differential scanning calorimetry curves of PEO (red), PCL (blue), and PTMC (green) with 0, 25 and 50 wt% LiTFSI.

2.4. Nyquist and Bode plots of SPEs with 50 wt% LiTFSI

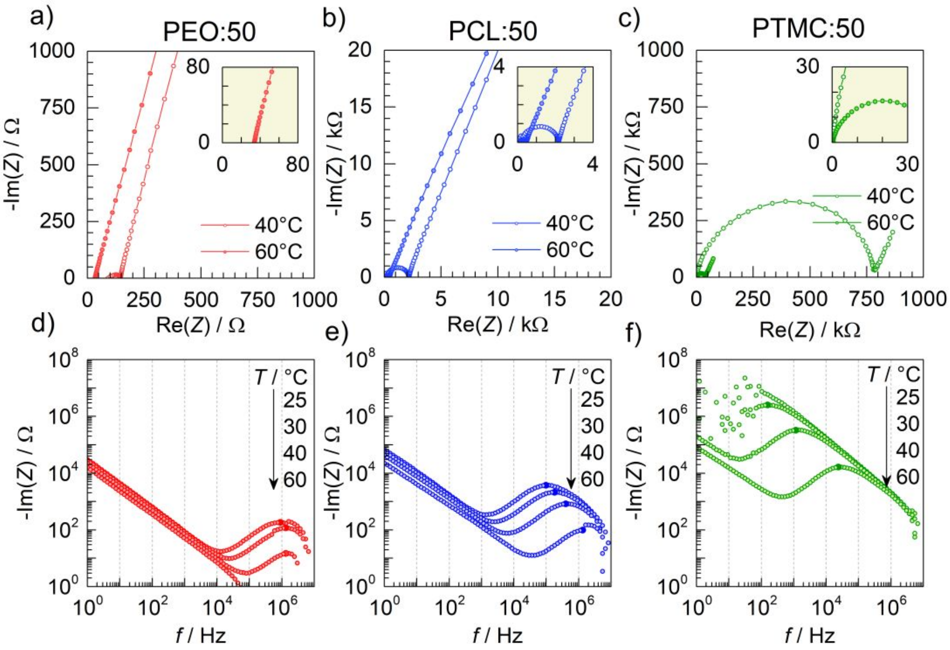

Figure S4. Nyquist and Bode plots of PEO:50 (red), PCL:50 (blue) and PTMC:50 (green) at temperatures ranging from 25 to 60 °C. The local maximum corresponding to the relaxation frequency for ion transport in the bulk polymer electrolyte have been marked in the Bode plots.

2.5. Equivalent circuit fitting parameters for SPEs and DLSPEs with 50 wt% LiTFSI

Table S10. Equivalent circuit fitting parameters for PEO with 50 wt% LiTFSI at temperatures ranging from 25 to 90 °C. The thickness and area of the film was 74 μm and 1.33 cm^2, respectively. The error percentages are listed in parentheses and errors exceeding 10% are marked in red.

T / K	***R*** $_{PEO}$ / Ω		***CPE*** $_{g, PEO}$ / F s^{1-p}		***CPE*** $_{dl}$ / F s^{1-p}		**σ** / Scm^{-1}
298.15	598.2	(1.06)	1.04×10^{-8}	(22.30)	5.68×10^{-6}	(9.38)	5.66×10^{-9}
303.15	367	(1.15)	1.98×10^{-8}	(30.41)	6.44×10^{-6}	(8.42)	1.38×10^{-8}
313.15	143.7	(0.19)			8.32×10^{-6}	(1.03)	1.25×10^{-7}
323.15	63.6	(0.21)			1.01×10^{-5}	(0.90)	5.80×10^{-6}
333.15	33.14	(0.23)			1.23×10^{-5}	(0.78)	6.30×10^{-5}
343.15	18.45	(0.30)			1.46×10^{-5}	(0.85)	1.35×10^{-4}
353.15	11.06	(0.39)			1.76×10^{-5}	(0.97)	2.48×10^{-4}
363.15	7.20	(0.56)			2.09×10^{-5}	(1.22)	4.17×10^{-4}

Table S11. Equivalent circuit fitting parameters for PCL with 50 wt% LiTFSI at temperatures ranging from 25 to 90 °C. The thickness and area of the film was 218 μm and 1.33 cm^2, respectively. The error percentages are listed in parentheses and errors exceeding 10% are marked in red.

T / K	***R*** $_{PCL}$ / Ω		***CPE*** $_{g, PCL}$ / F s^{1-p}		***CPE*** $_{dl}$ / F s^{1-p}		**σ** / Scm^{-1}
298.15	9164	(0.01)	6.69×10^{-10}	(2.99)	2.35×10^{-6}	(2.02)	1.79×10^{-6}
303.15	5273	(0.09)	5.46×10^{-10}	(3.97)	2.87×10^{-6}	(1.23)	3.11×10^{-6}
313.15	2147	(0.13)	3.05×10^{-10}	(13.05)	4.24×10^{-6}	(1.14)	7.63×10^{-6}
323.15	884.7	(0.21)	9.77×10^{-10}	(11.59)	6.47×10^{-6}	(1.54)	1.85×10^{-5}
333.15	440.1	(0.72)	4.32×10^{-9}	(25.70)	9.11×10^{-6}	(4.78)	3.72×10^{-5}
343.15	233	(0.24)			1.26×10^{-5}	(0.19)	7.03×10^{-5}
353.15	123.7	(0.28)			1.72×10^{-5}	(0.90)	1.33×10^{-4}
363.15	75.57	(0.27)			2.23×10^{-5}	(0.78)	2.17×10^{-4}

Table S12. Equivalent circuit fitting parameters for PTMC with 50 wt% LiTFSI at temperatures ranging from 25 to 90 °C. The thickness and area of the film was 105 μm and 1.33 cm^2, respectively. The error percentages are listed in parentheses and errors exceeding 10% are marked in red.

T / K	***R*** $_{PTMC}$ / Ω		***CPE*** $_{g, PTMC}$ / F s^{1-p}		***CPE*** $_{dl}$ / F s^{1-p}		**σ** / Scm^{-1}
298.15							
303.15	5.02×10^{6}	(2.10)	3.25×10^{-10}	(4.55)			1.57×10^{-9}
313.15	791880	(0.24)	3.94×10^{-10}	(1.56)	1.04×10^{-6}	(6.37)	9.97×10^{-9}
323.15	153640	(0.24)	4.49×10^{-10}	(2.94)	1.66×10^{-6}	(2.29)	5.14×10^{-8}
333.15	38365	(0.20)	3.33×10^{-10}	(5.11)	2.71×10^{-6}	(1.06)	2.06×10^{-7}
343.15	9904	(0.15)	3.91×10^{-10}	(4.91)	4.71×10^{-6}	(0.65)	7.97×10^{-7}
353.15	3748	(0.21)	4.31×10^{-10}	(7.07)	6.87×10^{-6}	(0.81)	2.11×10^{-6}
363.15	1565	(0.26)	5.97×10^{-10}	(10.20)	9.56×10^{-6}	(0.96)	5.04×10^{-6}

Table S13. Equivalent circuit fitting parameters for PEO-PCL with 50 wt% LiTFSI at temperatures ranging from 25 to 90 °C. The thickness and area of the film was 170 μm and 1.33 cm^2, respectively. The error percentages are listed in parentheses and errors exceeding 10% are marked in red.

T / K	***R*** $_{PEO+PCL}$ / Ω		***CPE*** $_{g, PEO+PCL}$ / F s^{1-p}		***R*** $_{pi}$ / Ω		***CPE*** $_{pi}$ / F s^{1-p}		***CPE*** $_{dl}$ / F s^{1-p}		**σ** / Scm^{-1}
298.15	43181	(1.26)	7.09×10^{-10}	(3.88)	49238	(1.26)	1.07×10^{-8}	(5.32)	1.17×10^{-6}	(0.55)	1.38×10^{-6}
303.15	19119	(0.96)	9.10×10^{-10}	(3.14)	16390	(1.24)	1.48×10^{-8}	(5.23)	1.37×10^{-6}	(0.38)	3.60×10^{-6}
313.15	5453	(1.12)	3.38×10^{-9}	(6.69)	1320	(7.54)	4.95×10^{-9}	(23.15)	1.27×10^{-6}	(0.37)	2.01×10^{-6}
323.15	316.6	(2.72)			902.5	(1.01)	1.11×10^{-9}	(7.56)	2.22×10^{-6}	(0.75)	1.05×10^{-5}
333.15	180.6	(14.56)			201.4	(13.48)	4.10×10^{-9}	(60.13)	3.44×10^{-6}	(8.30)	3.35×10^{-6}
					R $_{Total}$						
343.15					179.6	(0.12)			3.72×10^{-6}	(1.26)	7.12×10^{-5}
353.15					102.8	(0.27)			4.96×10^{-6}	(2.23)	1.24×10^{-4}
363.15					63.89	(0.38)			6.83×10^{-6}	(3.54)	2.00×10^{-4}

Table S14. Equivalent circuit fitting parameters for PEO-PTMC with 50 wt% LiTFSI at temperatures ranging from 25 to 90 °C. The thickness and area of the film was 172 μm and 1.33 cm², respectively. The error percentages are listed in parentheses and errors exceeding 10% are marked in red.

T / K	***R*** $_{Total}$ / Ω		***CPE*** $_{g, Total}$ / F s^{1-p}		***R*** $_{pi}$ / Ω		***CPE*** $_{pi}$ / F s^{1-p}		***CPE*** $_{dl}$ / F s^{1-p}		**σ** / Scm^{-1}
298.15	6734200	(3.04)	2.26×10^{-10}	(3.64)					3.90×10^{-7}	(17.16)	1.92×10^{-9}
303.15	1823200	(0.54)	3.70×10^{-10}	(2.02)					5.41×10^{-7}	(4.94)	7.09×10^{-9}
	R $_{PEO+PTMC}$		***CPE*** $_{g, PEO+PTMC}$								
313.15	285430	(2.33)	3.70×10^{-10}	(1.91)	56643	(12.26)	8.18×10^{-10}	(38.03)	9.14×10^{-7}	(2.55)	3.78×10^{-8}
323.15	61304	(1.72)	4.59×10^{-10}	(4.39)	11398	(8.86)	7.97×10^{-10}	(12.36)	1.54×10^{-6}	(0.89)	1.78×10^{-7}
333.15	12258	(8.94)	5.07×10^{-10}	(6.70)	3380	(32.52)	3.76×10^{-10}	(51.806)	2.81×10^{-6}	(0.48)	8.27×10^{-7}
	R $_{Total}$		***CPE*** $_{g, Total}$								
343.15	5035	(0.21)	7.08×10^{-10}	(6.81)					4.61×10^{-6}	(0.91)	2.57×10^{-6}
353.15	1922	(0.29)	1.14×10^{-9}	(10.09)					7.48×10^{-6}	(1.51)	6.73×10^{-6}
363.15	781.7	(0.23)	1.20×10^{-9}	(16.23)					1.12×10^{-5}	(2.05)	1.65×10^{-5}

Table S15. Equivalent circuit fitting parameters for PCL-PTMC with 50 wt% LiTFSI at temperatures ranging from 25 to 90 °C. The thickness and area of the film was 310 μm and 1.33 cm², respectively.

T / K	***R*** $_{Total}$ / Ω		***CPE*** $_{g, Total}$ / F s^{1-p}		***R*** $_{pi}$ / Ω		***CPE*** $_{pi}$ / F s^{1-p}		***CPE*** $_{dl}$ / F s^{1-p}		**σ** / Scm^{-1}
298.15	4522200	(0.75)	2.91×10^{-10}	(3.48)							5.15×10^{-9}
	R $_{PCL+PTMC}$		***CPE*** $_{g, PCL+PTMC}$								
303.15	999700	(1.80)	3.70×10^{-10}	(1.91)	260690	(7.43)	2.16×10^{-9}	(12.68)			1.85×10^{-8}
313.15	170550	(31.62)	4.59×10^{-10}	(4.39)	72084	(74.74)	9.50×10^{-10}	(26.50)	2.77×10^{-6}	(0.75)	9.61×10^{-8}
323.15	42173	(1.63)	5.07×10^{-10}	(6.70)	17622	(3.86)	4.05×10^{-10}	(6.61)	3.66×10^{-6}	(2.53)	3.90×10^{-7}
	R $_{Total}$		***CPE*** $_{g, Total}$								
333.15	15291	(0.20)	5.64×10^{-10}	(4.27)					5.42×10^{-6}	(1.08)	1.53×10^{-6}
343.15	5465	(0.26)	7.64×10^{-10}	(6.23)					7.45×10^{-6}	(1.32)	4.27×10^{-6}
353.15	2227	(0.23)	6.39×10^{-10}	(9.62)					1.05×10^{-6}	(1.15)	1.05×10^{-5}
363.15	973.7	(0.19)	7.39×10^{-10}	(13.88)					1.42×10^{-6}	(1.31)	2.39×10^{-5}

2.6. Total ionic conductivity of DLSPEs

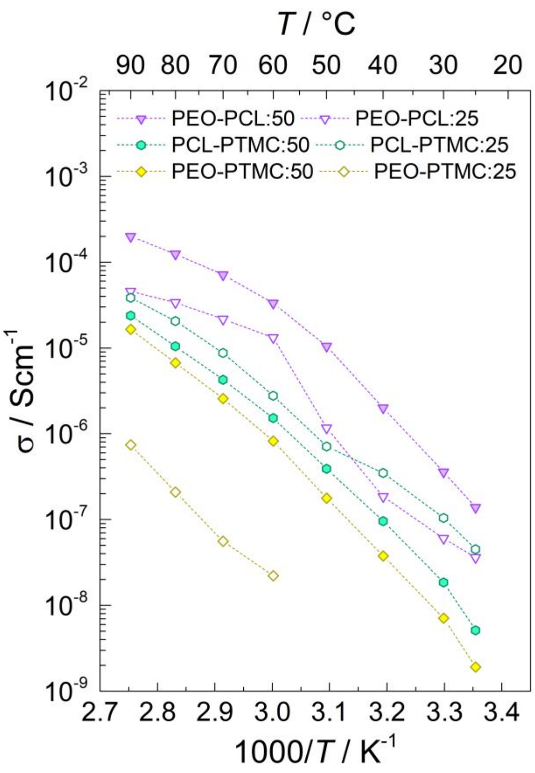

Figure S5. Total ionic conductivity of DLSPEs with 25 amd 50 wt% LiTFSI at temperatures ranging from 25 to 90 °C.

2.7. Morphology of the polymer–polymer interface in DLSPEs

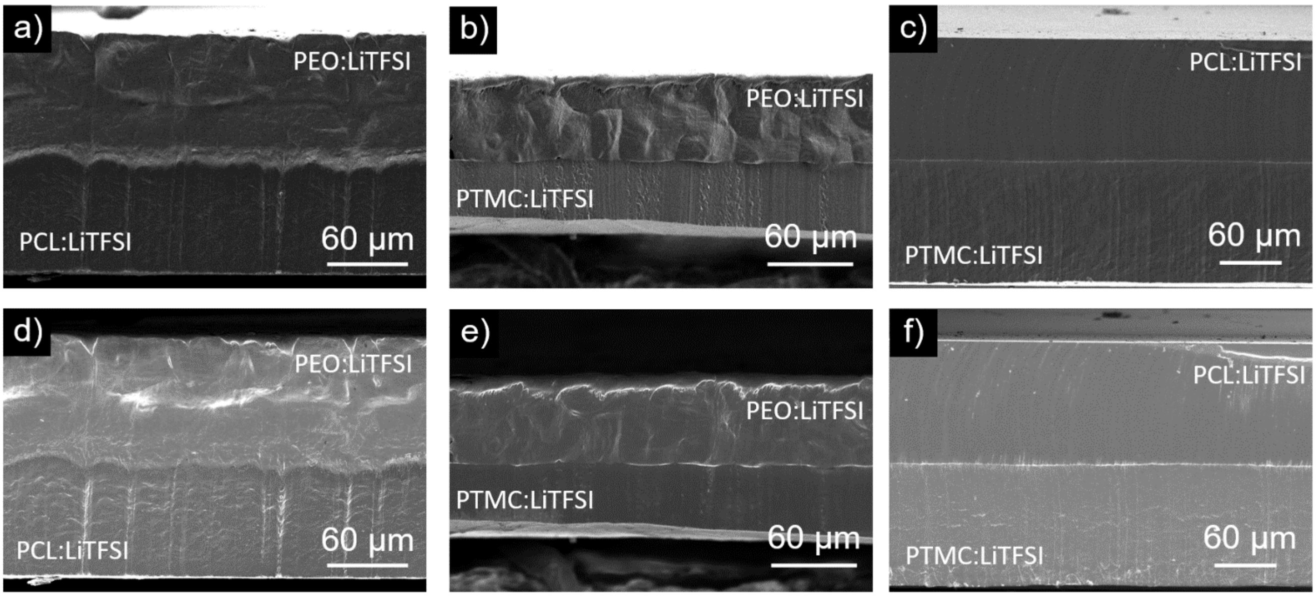

Figure S6. SEM micrographs of PEO-PCL:25 wt% LiTFSI (a and c) and PEO-PTMC:25 wt% LiTFSI (b and d) cross-sections. Top and bottom micrographs were taken using HE-SE2 and InLens detectors, respectively. The approximate total thickness of PEO-PCL:25, PEO-PTMC:25 and PCL-PTMC:25 was 171.2, 93.05 and 247.5 μm, respectively. The approximate thickness of the PEO layers in PEO:PCL:25 and PEO-PTMC:25 was 86.5 and 51.4 μm, respectively. The thickness of the PTMC layer in PCL-PTMC:25 was 124.8 μm.

3. References

(1) Sun, B.; Mindemark, J.; Edström, K.; Brandell, D. Polycarbonate-Based Solid Polymer Electrolytes for Li-Ion Batteries. *Solid State Ionics* **2014,** *262*, 738-742.

(2) Teran, A. A.; Tang, M. H.; Mullin, S. A.; Balsara, N. P. Effect of Molecular Weight on Conductivity of Polymer Electrolytes. *Solid State Ionics* **2011,** *203* (1), 18-21.

(3) Timachova, K.; Watanabe, H.; Balsara, N. P. Effect of Molecular Weight and Salt Concentration on Ion Transport and the Transference Number in Polymer Electrolytes. *Macromolecules* **2015,** *48* (21), 7882-7888.

(4) Mindemark, J.; Törmä, E.; Sun, B.; Brandell, D. Copolymers of Trimethylene Carbonate and ε-Caprolactone as Electrolytes for Lithium-Ion Batteries. *Polymer* **2015,** *63*, 91-98.
